# The Potential Risk Factors for Mortality in Patients After In-Hospital Cardiac Arrest: A Multicenter Study

**DOI:** 10.3389/fcvm.2021.630102

**Published:** 2021-03-16

**Authors:** Mei-Tzu Wang, Wei-Chun Huang, David Hung-Tsang Yen, En-Hui Yeh, Shih-Yuan Wu, Hsun-Hsiang Liao

**Affiliations:** ^1^Department of Critical Care Medicine, Kaohsiung Veterans General Hospital, Kaohsiung, Taiwan; ^2^Section of Cardiology, Department of Internal Medicine, Kaohsiung Veterans General Hospital, Kaohsiung, Taiwan; ^3^School of Medicine, National Yang-Ming University, Taipei, Taiwan; ^4^Department of Physical Therapy, Fooyin University, Kaohsiung, Taiwan; ^5^Department of Emergency Medicine, Taipei Veterans General Hospital, Taipei, Taiwan; ^6^Institute of Emergency and Critical Care Medicine, College of Medicine, National Yang-Ming University, Taipei, Taiwan; ^7^Joint Commission of Taiwan, New Taipei City, Taiwan

**Keywords:** intensive care unit, in-hospital cardiac arrest, overnight shift, patient-to-nurse ratio, survival, targeted temperature management

## Abstract

**Background and Purpose:** In-hospital cardiac arrest (IHCA) has high mortality rate, which needs more research. This multi-center study aims to evaluate potential risk factors for mortality in patients after IHCA.

**Methods:** Data for this study retrospectively enrolled IHCA patients from 14 regional hospitals, two district hospitals, and five medical centers between 2013 June and 2018 December. The study enrolled 5,306 patients and there were 2,871 patients in subgroup of intensive care unit (ICU) and emergency room (ER), and 1,894 patients in subgroup of general wards.

**Results:** As for overall IHCA patients, odds ratio (OR) for mortality was higher in older patients (OR = 1.69; 95% CI:1.33–2.14), those treated with ventilator (OR = 1.79; 95% CI:1.36–2.38) and vasoactive agents (OR = 1.88; 95% CI:1.45–2.46). Whereas, better survival was reported in IHCA patients with initial rhythm as ventricular tachycardia (OR = 0.32; 95% CI: 0.21–0.50) and ventricular fibrillation (OR = 0.26; 95% CI: 0.16–0.42). With regard to ICU and ER subgroup, there was no mortality difference among different nursing shifts, whereas for patients in general wards, overnight shift (OR = 1.83; 95% CI: 1.07–3.11) leads to poor outcome.

**Conclusion:** For IHCA patients, old age, receiving ventilator support and vasoactive agents reported poor survival. Overnight shift had poor survival for IHCA patients in general wards, despite no significance in overall and ICU/ER subgroups.

## Introduction

In-hospital cardiac arrest (IHCA) has high mortality rate (1). The majority of data are derived from the American Heart Association's Get With The Guidelines-Resuscitation (GWTG-R) registry, which reported 9 to 10 IHCA cases per 1,000 admissions from 2008 to 2017 (2, 3).

Despite successful resuscitation, only few resuscitated patients have good neurologic conditions at discharge (4). Targeted temperature management (TTM) after cardiac arrest remains the primary neuroprotective approach following cardiac arrest (5, 6).

Compared to other critical cardiovascular conditions, including stroke, myocardial infarction, and OHCA, IHCA has received little attention (1). Thus, this study was conducted to evaluate the potential risk factors for mortality in patients after IHCA.

## Methods

This cross-sectional study analyzed the IHCA data set of The Taiwan Clinical Performance Indicator (TCPI) system, which was founded by the Joint Commission of Taiwan (JCT) in 2011. The Human Research Committee of Kaohsiung Veterans General Hospital approved this study.

### Data Source and Study Population

**Figure 1:**
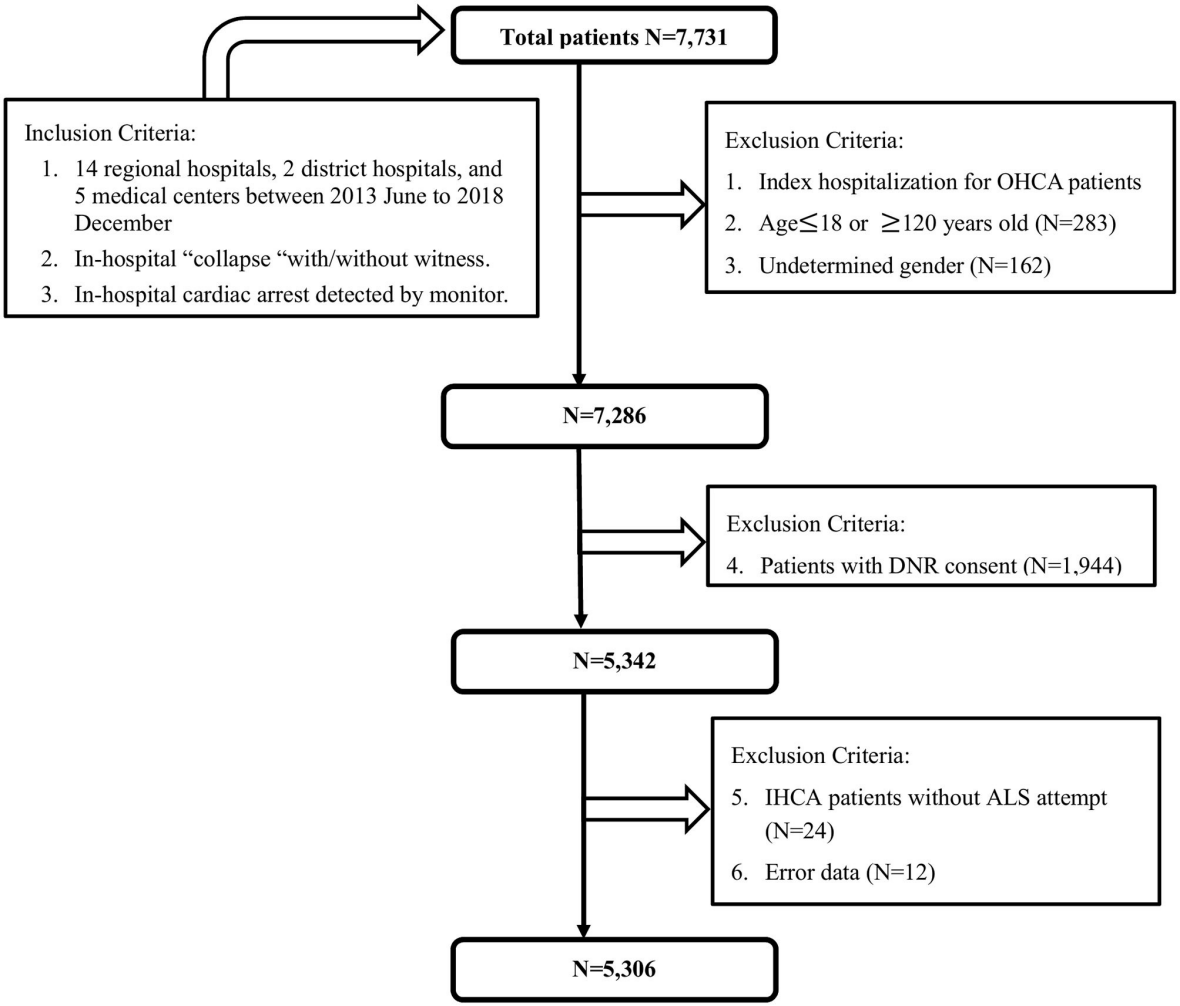
Flow-chart showing identification process of the study cohort.

This registered multicenter study retrospectively enrolled IHCA patients from 14 regional hospitals, two district hospitals, and five medical centers between June 2013 and December 2018. A total of 7,731 cases were included. We excluded patients with undetermined sex, age younger than 18 years, with a do-not-resuscitate (DNR) order, and those not receiving resuscitation. JCT staff supervised this registry and checked the numbers of IHCA patients to confirm that all IHCA patients in enrolled hospitals were included in this study. Finally, 5,306 patients were included in the analysis (Figure 1). The IHCA event locations were collected including intensive care unit (including coronary care units), emergent department, ordinary ward, examination room, postoperative recovery room, outpatient department, operating theater or coronary angiography laboratory and others. Total 54.1% of IHCA occurred in ICU/ER and 35.7% of IHCA occurred in general ward. Only 10.2% of IHCA occurred in examination room, postoperative recovery room, outpatient department, operating theater or coronary angiography laboratory, etc. Finally, there were 2,871 and 1,894 patients in the ICU/ER subgroup and general ward subgroup, respectively. Fetal arrhythmia was defined brady-arrhythmias or tachy-arrhythmias leading to sudden cardiac death (7). Ventricular tachycardia (VT) and ventricular fibrillation (VF) are kinds of fetal arrhythmia.

### Indications for TTM, Ventilator and Vasoactive Therapy

TTM has been recommended for post-resuscitation care of comatose patients after CA event (6, 8). Intubation and ventilator therapy are indicated for cardiac arrest patients not responsive, ineffective or not possible to bag-mask ventilation, and were needed for protected airway (9). The use of the vasopressors were recommended for the resuscitation of cardiac arrest patients in current guideline (10).

### Statistical Analysis

For data analysis, SAS software version 9.4 (SAS Institute, Inc., Cary, NC) was used. Percentile values were used to express categorical data, analyzed using the chi-square test. Multivariable logistic regression model was used to calculate odds ratio (OR) and associated 95% confidence intervals (95% CIs) for significant variables, including sex, age, hospital level, direct cause, and initial rhythm. A *P*-value <0.05 was considered statistically significant.

## Results

The patients' basic characteristics are listed in Table 1. The majority of the initially detected rhythms were pulseless electrical activity (PEA) (41.6%) and asystole (27.5%), and the leading cause of IHCA was fatal arrythmia (35.7%).

**Table 1:** Basic characteristics of in-hospital cardiac arrest patients in a multicenter cohort study.

**Variables**		**Total** **(*****N*** **=** **5,306)**		**Survival** **(*****N*** **=** **821)**		**Death** **(*****N*** **=** **4,485)**		***P*-value**
Sex	Male	3,335	(62.9%)	494	(60.2%)	2,841	(63.3%)	0.0836
	Female	1,971	(37.2%)	327	(39.8%)	1,644	(36.7%)	
Age	<70	2,537	(47.8%)	440	(53.6%)	2,097	(46.8%)	0.0003
	≥70	2,769	(52.2%)	381	(46.4%)	2,388	(53.2%)	
Hospital level	Medical center	2,588	(48.8%)	309	(37.6%)	2,279	(50.8%)	<0.0001
	Regional/district hospital	2,718	(51.2%)	512	(62.4%)	2,206	(49.2 %)	
Hospital volume for admission(*N* = average people per month)	*N* < 1,000	575	(10.8%)	79	(9.6%)	496	(11.1%)	<0.0001
	1,000 ≤ *N* < 2,000	1,070	(20.2%)	196	(23.9%)	874	(19.5%)	
	2,000 ≤ *N* < 3,000	1,027	(19.4%)	219	(26.7%)	808	(18.0%)	
	*N* ≥ 3,000	2,634	(49.6%)	327	(39.8%)	2,307	(51.4%)	
Hospital volume for emergency department(*N* = average people per month)	N < 3,000	324	(6.1%)	33	(4.0%)	291	(6.5%)	<0.0001
	3,000 ≤ *N* < 5,000	1,771	(33.4%)	335	(40.8%)	1,436	(32.0%)	
	5000 ≤ *N* < 7,000	1,454	(27.4%)	282	(34.4%)	1,172	(26.1%)	
	*N* ≥ 7,000	1,757	(33.1%)	171	(20.8%)	1,586	(35.4%)	
Total beds per hospital	<500	974	(18.4%)	145	(17.7%)	829	(18.5%)	0.0024
	500–1,000	1,600	(30.2%)	289	(35.2%)	1,311	(29.2%)	
	≥1,000	2,732	(51.5%)	387	(47.1%)	2,345	(52.3%)	
Event time	2013–2016	3,456	(65.1%)	459	(55.9%)	2,997	(66.8%)	<0.0001
Years	2017–2018	1,850	(34.9%)	362	(44.1%)	1,488	(33.2%)	
Months	March to May	1,334	(25.1%)	220	(26.8%)	1,114	(24.8%)	0.5994
	June to August	1,216	(22.9%)	177	(21.6%)	1,039	(23.2%)	
	September to November	1,247	(23.5%)	193	(23.5%)	1,054	(23.5%)	
	December to February	1,509	(28.4%)	231	(28.1%)	1,278	(28.5%)	
Office hour	Office hour	3,384	(63.8%)	547	(66.6%)	2,837	(63.3%)	0.0647
	Non-office hour	1,922	(36.2%)	274	(33.4%)	1,648	(36.7%)	
Shift	08:00~16:00	1,913	(36.1%)	337	(41.1%)	1,576	(35.1%)	<0.0001
	16:00~24:00	1,489	(28.1%)	274	(33.4%)	1,215	(27.1%)	
	24:00~08:00	1,904	(35.9%)	210	(25.6%)	1,694	(37.8%)	
Event location	Intensive care unit	1,866	(35.2%)	257	(31.3%)	1,609	(35.9%)	<0.0001
	Examination room	360	(6.8%)	89	(10.8%)	271	(6.0%)	
	Emergent department	1,005	(18.9%)	170	(20.7%)	835	(18.6%)	
	Ordinary ward	1,894	(35.7%)	273	(33.3%)	1,621	(36.1%)	
	Others	181	(3.4%)	32	(3.9%)	149	(3.3%)	
Witness	With	4,560	(85.9%)	781	(95.1%)	3,779	(84.3%)	<0.0001
With treatment/ procedure before ALS		4,194	(79.0%)	694	(84.5%)	3,500	(78.0%)	<0.0001
Intravascular catheter		3,816	(71.9%)	640	(78.0%)	3,176	(70.8%)	<0.0001
Intravascular medication		2,932	(55.3%)	455	(55.4%)	2,477	(55.2%)	0.9191
Electrocardiogram monitor		2,708	(51.0%)	448	(54.6%)	2,260	(50.4%)	0.0277
Intubation		1,615	(30.4%)	213	(25.9%)	1,402	(31.3%)	0.0023
Ventilator		1,513	(28.5%)	200	(24.4%)	1,313	(29.3%)	0.0041
Intracardiac pacemaker or defibrillator		105	(2.0%)	19	(2.3%)	86	(1.9%)	0.4530
Arterial line		747	(14.1%)	109	(13.3%)	638	(14.2%)	0.4724
Cause of IHCA								
Fatal arrythmia		1,893	(35.7%)	366	(44.6%)	1,527	(34.1%)	<0.0001
Hypotension		658	(12.4%)	56	(6.8%)	602	(13.4%)	
Respiratory depression		792	(14.9%)	118	(14.4%)	674	(15.0%)	
Metabolism		180	(3.4%)	21	(2.6%)	159	(3.6%)	
Myocardial ischemia/infarction		389	(7.3%)	55	(6.7%)	334	(7.5%)	
Others		1,394	(26.3%)	205	(25.0%)	1,189	(26.5%)	
ALS item								
Chest compression		4,696	(88.5%)	727	(88.6%)	3,969	(88.5%)	0.9634
Electrical discharge		1,118	(21.1%)	309	(37.6%)	809	(18.0%)	<0.0001
Airway management		3,422	(64.5%)	543	(66.1%)	2,879	(64.2%)	0.2838
Vasoactive agents		4,132	(77.9%)	579	(70.5%)	3,553	(79.2%)	<0.0001
Vital sign before ALS								
Conscious	With	597	(11.3%)	102	(12.4%)	495	(11.0%)	0.3430
	Without	4,377	(82.5%)	674	(82.1%)	3,703	(82.6%)	
Respiration	With	1,564	(29.5%)	250	(30.5%)	1,314	(29.3%)	0.4768
	Without	3,384	(63.8%)	523	(63.7%)	2,861	(63.8%)	
Pulse	Present	1,382	(26.1%)	226	(27.5%)	1,156	(25.8%)	0.5364
	Absent	3,565	(67.2%)	543	(66.1%)	3,022	(67.4%)	
Initial rhythm								
Ventricular fibrillation		276	(5.2%)	94	(11.5%)	182	(4.1%)	<0.0001
Ventricular tachycardia		548	(10.3%)	179	(21.8%)	369	(8.2%)	
Asystole		1,457	(27.5%)	155	(18.9%)	1,302	(29.1%)	
Pulseless electrical activity		2,205	(41.6%)	267	(32.5%)	1,938	(43.2%)	
Bradycardia		717	(13.5%)	102	(12.4%)	615	(13.7%)	
Perfusing rhythm		94	(1.8%)	22	(2.7%)	72	(1.6%)	
Start in-hospital resuscitation teams	Yes	2,148	(67.9%)	392	(67.6%)	1,756	(67.9%)	0.8822
Reason of CPR termination	Death	1,378	(26.0%)	0	(0.0%)	1,378	(30.7%)	<0.0001
	Medical futility	498	(9.4%)	13	(1.6%)	485	(10.8%)	
	DNR	949	(17.9%)	15	(1.8%)	934	(20.8%)	
	ROSC	2,447	(46.1%)	787	(95.9%)	1,660	(37.0%)	
	Mechanical support (ECMO)	34	(0.6%)	6	(0.7%)	28	(0.6%)	
Targeted temperature management	With	24	(0.5%)	9	(1.1%)	15	(0.3%)	0.0028

*ALS, advanced life support; CPR, cardiopulmonary resuscitation; DNR, do-not-resuscitate; ECMO, Extracorporeal Membrane Oxygenation; IHCA, in-hospital cardiac arrest; ROSC, Return of spontaneous circulation*.

**Figure 2:**
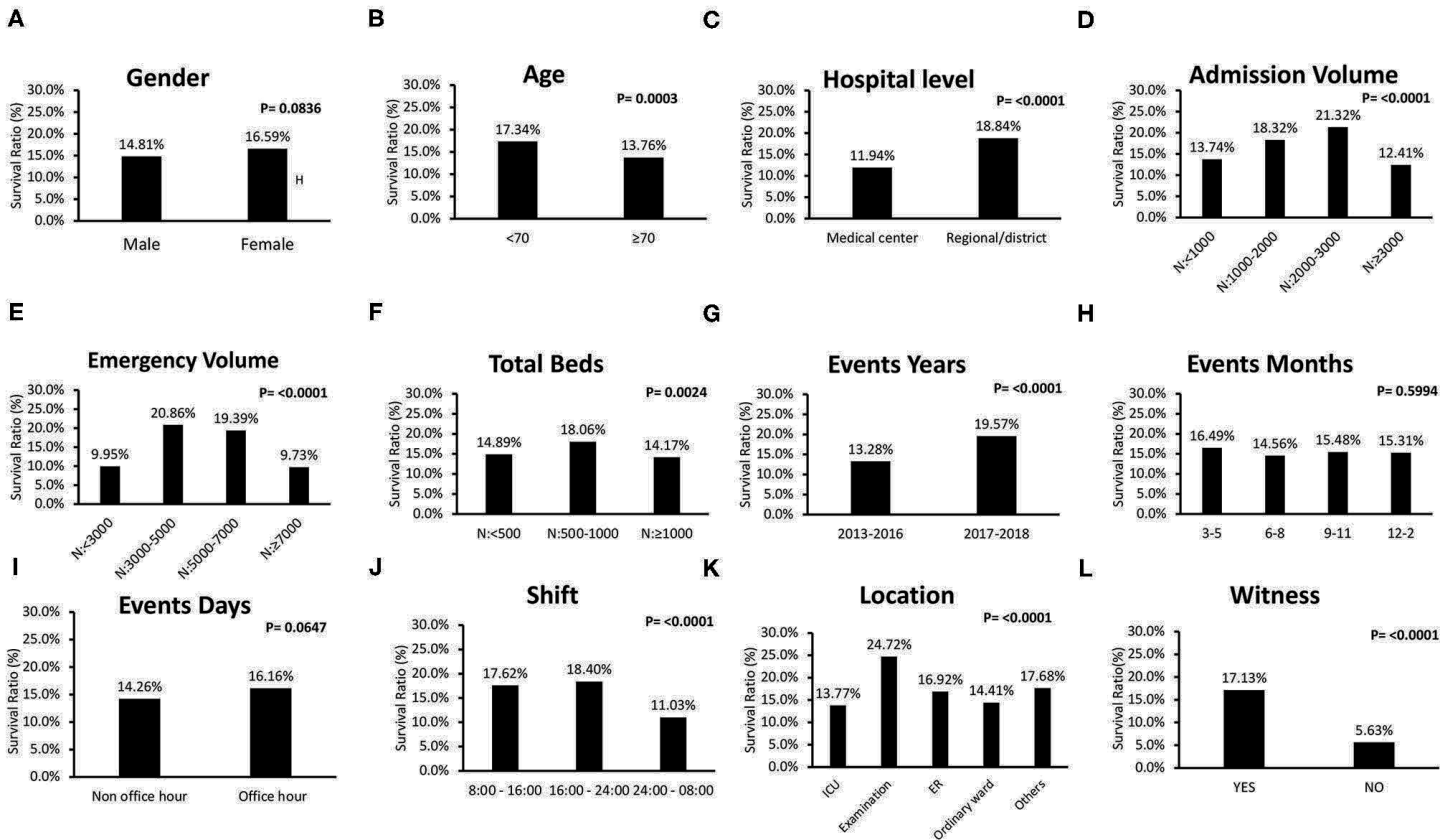
Comparisons of survival rate of individual clinical characteristic of in-hospital-cardiac-arrest (IHCA) patients. **(A)** There was no sex difference in survival (male: 14.8%; female: 16.6%, P = 0.0836). **(B–F)** Factors for poor survival included old age (<70 years: 17.3%; ≥70 years: 13.8%, P = 0.0003), medical center (medical center: 11.9%; regional/district hospital: 18.8%, P < 0.0001), large hospital volume for admission with >3,000 patients per month on average, large hospital volume for emergency room with >7,000 patients per month on average, and total beds >1,000. **(G)** Events occurring in the recent year had better outcomes. **(H)** There was no difference in IHCA outcome among different months. **(I)** There was no difference in IHCA outcome between events occurring during office and non-office hours. **(J)** Events occurring between 24:00 and 08:00 showed poor outcomes. (K) Event location at intensive care unit (ICU) showed poor outcomes, whereas, event location at examination room presented better outcomes (ICU: 13.8%; examination room: 24.7%; emergent department: 16.9%; ordinary ward: 14.4%; others: 17.7%, P < 0.000 1). **(L)** Better survival was reported in patients with a witness (with: 17.1% without: 5.6%, P < 0.0001).

Survival ratios of IHCA patients according to basic characteristics are shown in Figure 2. There was no sex difference in survival ratios (Figure 2A). Factors for poor survival included old age (Figure 2B), medical center (Figure 2C), large hospital volume for admission with >3,000 patients per month on average (Figure 2D), and total beds >1,000 (Figure 2F). Events occurring between 24:00 and 08:00 had poor outcomes (Figure 2J). Events occurring at the ICU showed poor outcomes, whereas events at the examination room showed better outcomes (Figure 2K). Moreover, better survival was reported in patients with a witness during the attack (Figure 2L).

**Figure 3:**
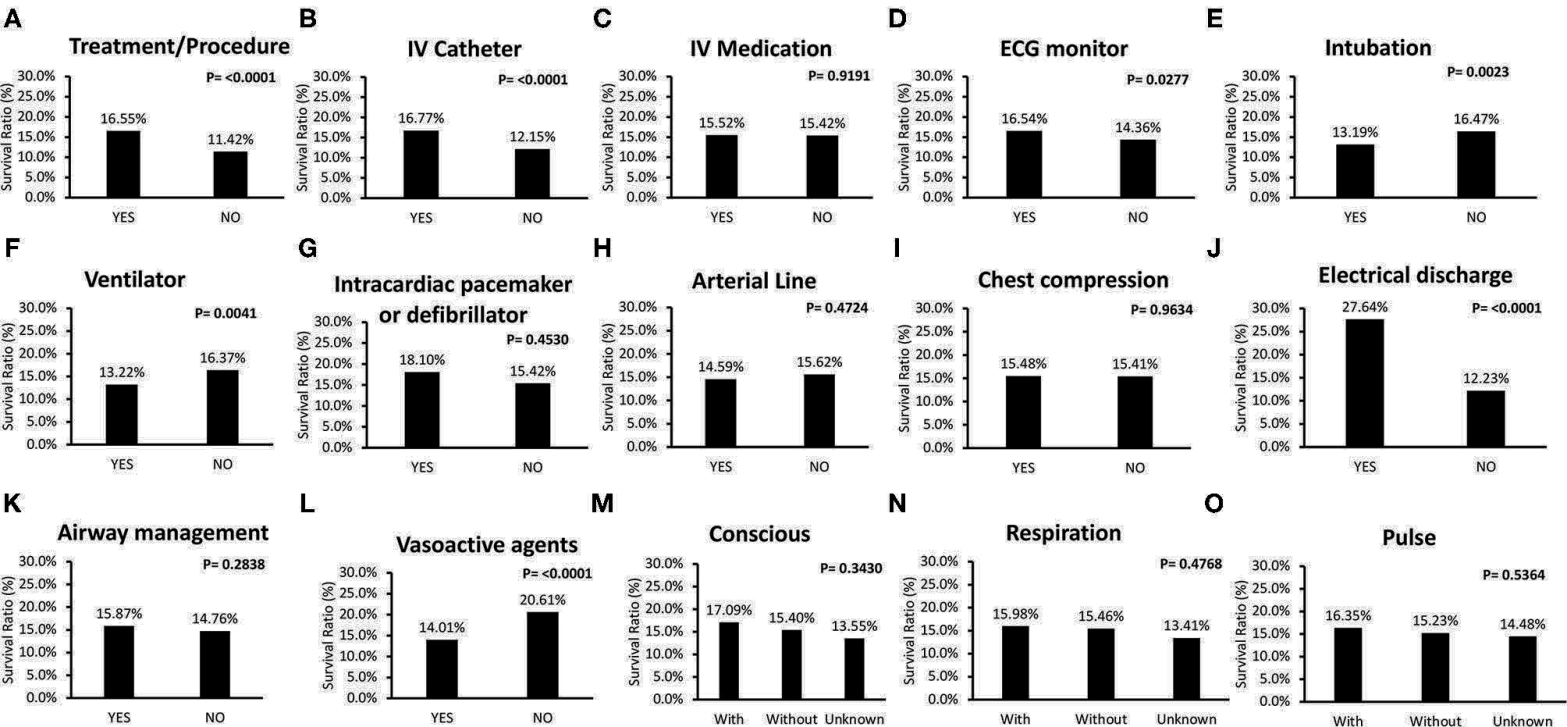
Comparisons of survival rate of treatment or procedure before advanced life support (ALS) and ALS associated items for in-hospital-cardiac-arrest (IHCA) patients. **(A,B,D–F)** Treatment or procedure contributed to better outcome, including intravascular (IV) catheter, electrocardiogram (ECG) monitor, intubation, and ventilator. **(C)** IV medication had no contribution to survival. **(G,H)**. There were no survival differences for IHCA patients to have intracardiac pacemaker or defibrillator, and arterial line before ALS. **(I,K)** Regarding ALS order, chest compression and airway management did not affect overall IHCA survival rate. **(J,L)** Patients received electrical discharge (with electrical discharge: 27.6%; without electrical discharge: 12.2%, P < 0.0001) and not be prescribed with vasoactive agents also presented better outcome (with vasoactive agents: 14.0%; without vasoactive agents: 20.6%, P < 0.0001) had better survival. **(M–O)** Vital sign before ALS, such as conscious, reparation, and pulse did not affect overall survival.

**Figure 4:**
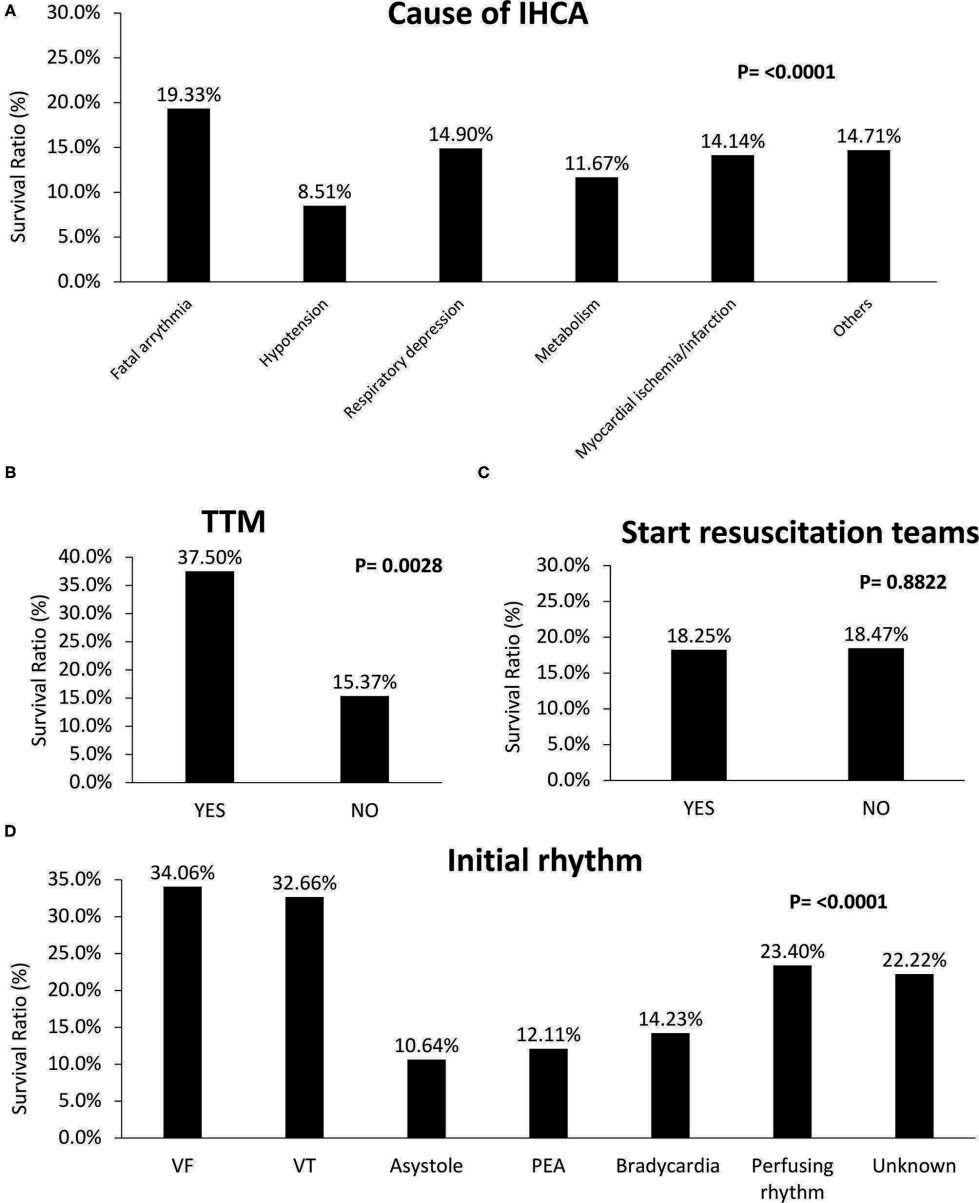
Comparisons of survival rate of different causes and initial rhythm of in-hospital-cardiac-arrest (IHCA) and other specific managements. **(A)** Fatal arrhythmia as the cause of IHCA had better survival. **(B)** Patients who underwent TTM (with: 37.5%; without TTM: 15.4%, P = 0.0028) had better outcomes. **(C)** Starting in-hospital resuscitation teams had no benefit on survival (with: 18.3%; without: 18.5%, P = 0.8822). **(D)** Initially detected ventricular fibrillation (VF) revealed better survival rates (VF: 34.1%; ventricular tachycardia (VT): 32.7%; asystole: 10.6%; PEA: 12.1%; bradycardia: 14.2%; perfusing rhythm: 23.4%; unknown: 22.2%, P < 0.0001).

Figure 3 includes survival ratios of treatment or procedure before ALS and ALS-associated items. The survival ratio with different causes of IHCA and other managements are reported in Figure 4. Fatal arrhythmia as the cause of IHCA had better survival (Figure 4A). Patients who underwent TTM had better outcomes (Figure 4B). Initially-detected ventricular fibrillation (VF) was associated with better survival ratios (Figure 4D). Moreover, there was no difference in survival between patients with and without in-hospital resuscitation teams (Figure 4C).

The basic characteristics of the general ward and ICU/ER subgroups are reported in Table 2. The general ward subgroup had more patients aged >70 years and more IHCA events occurring during overnight shifts. The ICU/ER subgroup had more IHCA events occurring during office hours, more witnessed IHCA patients, and more patients receiving treatment or procedure before ALS. Moreover, the proportion of starting in-hospital resuscitation teams was higher in the general subgroup.

**Table 2:** Basic characteristics of in-hospital cardiac arrest patients in subgroups of general wards and intensive care unit/emergency room.

**Variables**		**General wards** **(*****N*** **=** **1,894)**		**ICU/ER** **(*****N*** **=** **2,871)**		***P*-value**
Sex	Male	1,181	(62.4%)	1,847	(64.3%)	0.1650
	Female	713	(37.7%)	1,024	(35.7%)	
Age	<70	797	(42.1%)	1,480	(51.6%)	<0.0001
	≥70	1,097	(57.9%)	1,391	(48.5%)	
Hospital level	Medical center	904	(47.7%)	1,509	(52.6%)	0.0025
	Regional/district hospital	990	(52.3%)	1,362	(47.4%)	
Hospital volume for admission(*N* = average people per month)	*N* < 1,000	176	(9.3%)	377	(13.1%)	<0.0001
	1,000 ≤ *N* < 2,000	359	(19.0%)	621	(21.6%)	
	2,000 ≤ *N* < 3,000	401	(21.2%)	381	(13.3%)	
	*N* ≥ 3,000	958	(50.6%)	1492	(52.0%)	
Hospital volume for emergency department(*N* = average people per month)	*N* < 3,000	217	(11.5%)	398	(13.9%)	0.1466
	3,000 ≤ *N* < 5,000	531	(28.0%)	799	(27.8%)	
	5,000 ≤ *N* < 7,000	498	(26.3%)	704	(24.5%)	
	*N* ≥ 7,000	648	(34.2%)	979	(34.1%)	
Total beds per hospital	<500	266	(14.0%)	666	(23.2%)	<0.0001
	500–1,000	723	(38.2%)	730	(25.4%)	
	≥1,000	905	(47.8%)	1,475	(51.4%)	
Event time						
Years	2013–2016	1,187	(62.7%)	1891	(65.9%)	0.0241
	2017–2018	707	(37.3%)	980	(34.1%)	
Months	March to May	461	(24.3%)	723	(25.2%)	0.1264
	June to August	468	(24.7%)	637	(22.2%)	
	September to November	421	(22.2%)	699	(24.4%)	
	December to February	544	(28.7%)	812	(28.3%)	
Office hour	Office hour	1,118	(59.0%)	1897	(66.1%)	<0.0001
	Non office hour	776	(41.0%)	974	(33.9%)	
Shift	08:00~16:00	601	(31.7%)	1046	(36.4%)	<0.0001
	16:00~24:00	501	(26.5%)	848	(29.5%)	
	24:00~08:00	792	(41.8%)	977	(34.0%)	
Witness	With	1,569	(82.8%)	2,511	(87.5%)	<0.0001
With treatment/ procedure before ALS		1,454	(76.8%)	2,318	(80.7%)	0.0010
Intravascular catheter		1,314	(69.4%)	2,139	(74.5%)	0.0001
Intravascular medication		828	(43.7%)	1,835	(63.9%)	<0.0001
Electrocardiogram monitor		430	(22.7%)	1,985	(69.1%)	<0.0001
Intubation		129	(6.8%)	1,324	(46.1%)	<0.0001
Ventilator		117	(6.2%)	1,248	(43.5%)	<0.0001
Intracardiac pacemaker or defibrillator		12	(0.6%)	75	(2.6%)	<0.0001
Arterial line		19	(1.0%)	643	(22.4%)	<0.0001
Cause of IHCA						
Fatal arrythmia		528	(27.9%)	1,214	(42.3%)	<0.0001
Hypotension		152	(8.0%)	446	(15.5%)	
Respiratory depression		451	(23.8%)	286	(10.0%)	
Metabolism		49	(2.6%)	113	(3.9%)	
Myocardial ischemia/infarction		104	(5.5%)	205	(7.1%)	
Others		610	(32.2%)	607	(21.1%)	
ALS item						
Chest compression		1,678	(88.6%)	2,523	(87.9%)	0.4535
Electrical discharge		304	(16.1%)	658	(22.9%)	<0.0001
Airway management		1,490	(78.7%)	1,576	(54.9%)	<0.0001
Vasoactive agents		1,384	(73.1%)	2,338	(81.4%)	<0.0001
Vital sign before ALS						
Conscious	With	187	(9.9%)	338	(11.8%)	0.0002
	Without	1,615	(85.3%)	2,325	(81.0%)	
Respiration	With	415	(21.9%)	990	(34.5%)	<0.0001
	Without	1,377	(72.7%)	1,659	(57.8%)	
Pulse	Present	429	(22.7%)	806	(28.1%)	<0.0001
	Absent	1,361	(71.9%)	1,847	(64.3%)	
Initial rhythm						
Ventricular fibrillation		72	(3.8%)	171	(6.0%)	<0.0001
Ventricular tachycardia		109	(5.8%)	366	(12.8%)	
Asystole		794	(41.9%)	514	(17.9%)	
Pulseless electrical activity		706	(37.3%)	1293	(45.0%)	
Bradycardia		165	(8.7%)	489	(17.0%)	
Perfusing rhythm		48	(2.5%)	38	(1.3%)	
Start in-hospital resuscitation teams	Yes	1,051	(55.5%)	863	(30.1%)	<0.0001
Reason of CPR termination	Death	479	(25.3%)	751	(26.2%)	0.0016
	Medical futility	164	(8.7%)	289	(10.1%)	
	DNR	386	(20.4%)	504	(17.6%)	
	ROSC	863	(45.6%)	1,305	(45.5%)	
	Mechanical support	2	(0.1%)	22	(0.8%)	
Targeted temperature management		7	0.37%	12	0.42%	0.7954

*ALS, advanced life support; CPR, cardiopulmonary resuscitation; DNR, do not resuscitate; IHCA, in-hospital cardiac arrest; ROSC, Return of spontaneous circulation*.

In the multivariable logistic regression model, OR for mortality was higher in older patients, those receiving ventilator support before ALS, and those receiving vasoactive agents during ALS in the overall IHCA patient group (Table 3). On the contrary, patients with respiratory depression as the cause of IHCA, rather than hypotension showed better outcomes. In the ICU/ER subgroup (Table 3), better survival was reported in patients with respiratory depression rather than hypotension as the cause of IHCA, and patients with initially detected VT and VF had better outcome. For the general ward subgroup, patients with older age and attacks occurring between 24:00 and 08:00 (compared to 08:00–17:00) had poor outcomes. Moreover, initial rhythms of VT and VF were shown to reduce the mortality risk in IHCA patients in overall, ICU/ER and general ward subgroups.

**Table 3:** Multivariable logistic regression model on survival of IHCA patients. (*N* = 5,306).

	**Overall** ** (*****N*** **=** **5,306)**		**ICU and ER** ** (*****N*** **=** **2,871)**		**General Ward** ** (*****N*** **=** **1,894)**	
**Variables**	**Adjusted OR**	***P*-value**	**Adjusted OR**	***P*-value**	**Adjusted OR**	***P*-value**
	**(95% CI)**		**(95% CI)**		**(95% CI)**	
Male	1.26 (1.00–1.60)	0.0525	1.40 (1.01–1.95)	0.0456	1.18 (0.78–1.81)	0.4335
Age ≥70	1.69 (1.33–2.14)	<0.0001	1.48 (1.07–2.06)	0.0196	2.24 (1.47–3.42)	0.0002
Hospital level-Medical center	1.53 (1.12–2.08)	0.0072	1.57 (1.02–2.43)	0.0412	1.27 (0.71–2.26)	0.4238
Non-office hour	1.27 (0.99–1.61)	0.0568	1.15 (0.82–1.62)	0.4083	1.27 (0.82–1.95)	0.2847
Shift (Reference: 08:00~17:00)						
24:00~08:00	1.24 (0.92–1.66)	0.1585	0.59 (0.32–1.10)	0.0966	1.83 (1.07–3.11)	0.0268
17:00~24:00	0.81 (0.62–1.06)	0.1210	0.67 (0.40–1.12)	0.1263	0.97 (0.60–1.58)	0.9028
With witness	0.43 (0.16–1.11)	0.0803	1.02 (0.10–11.06)	0.9849	0.47 (0.16–1.41)	0.1783
Treatment/ procedure before ALS						
Intravascular catheter	0.93 (0.67–1.30)	0.6763	0.65 (0.38–1.12)	0.1223	1.14 (0.64–2.04)	0.6604
Intravascular medication	1.31 (1.00–1.70)	0.0463	1.72 (1.18–2.52)	0.0047	1.07 (0.67–1.69)	0.7826
Ventilator	1.79 (1.36–2.38)	<0.0001	1.95 (1.36–2.79)	0.0003	1.03 (0.43–2.50)	0.9467
Intracardiac pacemaker or defibrillator	1.99 (0.94–4.19)	0.0706	1.97 (0.80–4.87)	0.1403	0.74 (0.07–8.12)	0.8055
Cause (Reference: Hypotension)						
Myocardial ischemia/infarction	0.91 (0.56–1.47)	0.6945	0.68 (0.33–1.40)	0.2994	1.44 (0.52–3.97)	0.4798
Metabolism	0.69 (0.39–1.23)	0.2070	0.39 (0.18–0.83)	0.0149	1.01 (0.30–3.40)	0.9846
Respiratory depression	0.59 (0.40–0.87)	0.0071	0.37 (0.21–0.67)	0.0010	0.72 (0.37–1.42)	0.3418
Fatal arrythmia	0.56 (0.39–0.82)	0.0026	0.39 (0.23–0.67)	0.0005	0.56 (0.27–1.16)	0.1195
ALS order						
Chest compression	1.06 (0.73–1.54)	0.7652	1.24 (0.75–2.04)	0.3991	1.51 (0.70–3.24)	0.2921
Electrical discharge	0.88 (0.64–1.20)	0.4002	0.87 (0.56–1.34)	0.5240	1.08 (0.61–1.92)	0.7988
Airway management	0.96 (0.74–1.26)	0.7883	0.92 (0.64–1.34)	0.6753	0.90 (0.53–1.54)	0.7064
Vasoactive agents	1.88 (1.45–2.46)	<0.0001	1.77 (1.22–2.59)	0.0030	1.74 (1.07–2.83)	0.0250
With Respiration	0.93 (0.72–1.22)	0.6086	0.88 (0.61–1.27)	0.5027	0.93 (0.55–1.58)	0.7989
Initial rhythm (Reference: Asystole)						
Perfusing rhythm	0.71 (0.32–1.55)	0.3879	0.46 (0.12–1.79)	0.2597	1.10 (0.35–3.47)	0.8652
Bradycardia	0.62 (0.42–0.91)	0.0156	0.70 (0.39–1.27)	0.2417	0.63 (0.32–1.26)	0.1923
PEA	0.79 (0.56–1.12)	0.1811	0.84 (0.48–1.46)	0.5302	0.84 (0.49–1.45)	0.5393
Ventricular Tachycardia	0.32 (0.21–0.50)	<0.0001	0.32 (0.16–0.62)	0.0007	0.28 (0.12–0.67)	0.0044
Ventricular Fibrillation	0.26 (0.16–0.42)	<0.0001	0.24 (0.11–0.51)	0.0002	0.34 (0.15–0.81)	0.0149
Start in-hospital resuscitation teams	1.21 (0.93–1.56)	0.1512	1.27 (0.90–1.81)	0.1781	0.59 (0.27–1.28)	0.1781

## Discussion

This multicenter cohort study analyzed the association between IHCA patients and healthcare-related risk factors. Compared to the previous GWTG-R cohort data from the United States (U.S.) involving IHCA patients with a mean age of 66 years, our patients' mean age was 68 years and 62.9% of our patients were male (Table 1), whereas in the GWTG-R, 58% were male. The presenting rhythm in the GWTG-R was most often non-shockable (81%), which was comparable to our study finding showing that 82.6% of the patients had a non-shockable rhythm (1, 2). Survival to hospital discharge was ~25% in GWTG-R, whereas in our study, this was 15.5% (2). Previous study had a 7-year follow-up between 2000 and 2007, with 507 medical/surgical participating hospitals, and total 86,748 IHCA patients. It indicated lower survival during night hours in comparison with event occurred during day/evening hours (11). In comparison with this large sample size, our study followed 6 years, and had relatively small sample size with only 7,731 cases, however, our study analyzed the differences between survival and death subgroups to get information about the potential risk factors for IHCA mortality, in addition to the focus on the association between survival and event time. Preceding evidence indicated a 3% increase in in-hospital survival rates among IHCA patients between 2000 and 2004 (12). Moreover, advanced cardiac life support (ACLS) training and adherence contributed to better outcome of IHCA patients (13). Whereas, in comparison to previous study (11), rate of survival to discharge in overnight shift was not improved (14.7 vs. 11.03%), which might be attributed to hospital level and disease severity.

### Old Age Is a Predictor of Worse Overall Outcome

Age is one of the predictors of the Cardiac Arrest Survival Post Resuscitation In-hospital Score (CASPRI) score. A previous study indicated that increased age is associated with poor survival, especially for patients aged >70 years (1, 14). One single-center analysis of IHCA outcomes indicated that younger patients were more likely to survive the initial IHCA (15), which supported our study finding that age ≥70 years (OR= 1.69; 95% CI:1.33–2.14) (Table 3) was a predictor of worse overall outcome.

### Overnight Shift in General Wards Increased Mortality Risk of IHCA Patients

The patient-to-nurse ratio should be <9:1 in medical centers, 12:1 in regional hospitals, and 15:1 in district hospitals, according to Taiwan's local medical law standards. However, in Taiwan, the actual patient-to-nurse ratio is approximately 10–11:1 in general wards for day shifts and 20–30:1 for night shifts. This workload is five times higher than that of institutions in Europe or the U.S (16, 17).

Previous research reported 31% mortality for a 8:1 ratio for patients within 30 days of hospitalization (18, 19). Our study reported that overnight shift was associated with increased mortality of IHCA patients in general wards, which could be attributed to the highly disproportionate patient-to-nurse ratios and overload for overnight nursing staff in general wards (20). Previous study with a large sample size with 86,748 IHCA patients (58, 593 cases during day/evening hours; 28,155 cases during night hours) indicated lower survival during night hours in comparison with event occurred during day/evening hours (11), which supported our result.

### Initially-Detected VT/VF and Respiratory Depression as the Direct Cause of IHCA had Better Survival

Compared to all rhythms and non-shockable rhythms, VF and pulseless VT showed better survival and were two to three times more likely to survive to hospital discharge (1, 21). Higher prevalence of respiratory insufficiency was shown if the duration of preceding hospitalization was longer (22). Pre-existing data indicated that attempts at intubation may delay timely defibrillation and interruption in chest compressions, contributing to poor outcomes (23). However, in patients with respiratory depression as the cause of IHCA, intubation was not associated with worse survival (24).

Our study reported that patients with respiratory depression as the cause of IHCA had better survival in overall cohort and ICU/ER subgroup, but not in general ward subgroup. This result might be due to the fact that predictable or avoidable cardiac arrest may occur if patients had respiratory depression as the direct cause of IHCA events, and whether timely intubation affect survival.

### IHCA Patients With a Witness Showed Better Survival, and No Survival Difference Was Noted Between Patients With and Without In-Hospital Resuscitation Teams

Many IHCA cases are considered preventable or avoidable, and witnessed IHCA events are associated with improved outcomes (25, 26). Our study reported that witnessed IHCA patients in the ICU/ER subgroup did not have better outcomes, whereas the overall cohort and general ward subgroup showed better outcomes. This difference could be attributed to the existence of well-monitored settings and alarm systems in ICU/ER to offer timely response, despite the absence of a witness.

Early detected cardiac arrest events in monitored settings and early warning systems are associated with improved outcomes (27, 28). Event location at an examination room showed better outcomes in our study (Figure 2K). In our study, there was no survival difference between patients with and without in-hospital resuscitation teams (Figure 4C) (Table 3). This result is the contribution of well-trained staff, with sufficient skills to respond to possible preventable IHCA events in usual care.

### Study Limitations

First, our data were acquired from a unified medical record sheet filled out by professional practitioners, and information on comorbidities, past medical history, and underlying diseases were unavailable. Thus, post-resuscitation survival predictor such as CASPRI score could not be calculated. Second, detailed information such as definite collapse time, vital sign before resuscitation, and initial rhythm at collapse were not shown if IHCA events occurred in situations without monitor or witness. Moreover, resuscitation time, other interventions or devices and additional medical therapy were not documented. Finally, this was a multicenter, registered study, thus, future prospective randomized studies are required to confirm our findings. Finally, patients who underwent TTM had better outcomes, and this result was associated with the impact of TTM on post-resuscitation development rather than selection bias. However, this study enrolled IHCA patients between 2013 June and 2018 December, whereas national health insurance in Taiwan did not covered TTM until 2018 June. Selection bias might exist once life expectancy and economic issue were considered, however, there was no difference in terms of proportion of hypotension as cause of IHCA regardless receiving TTM or not (with: 16.67%; without: 12.38%, *P* = 0.5293). Furthermore, compared with previous study more than one decade ago, despite the improvement in ACLS training, rate of survival to discharge in overnight shift was not improved, which might be attributed to hospital level and disease severity. However, the evaluation of disease severity was our limitation.

### Study Strengths

This large registered multicenter study for adult IHCA patients enrolled different hospital levels and evaluated potential risk factors for mortality in patients after IHCA. Previous large sample size study focused on the association between survival and event time, thus it divided IHCA patients into events occurred during night hours and during day/evening hours (1). Our study analyzed the differences between survival and death subgroups to get information about the potential risk factors for IHCA mortality. Moreover, comparison between general wards and ICU/ER subgroups also strengthened the impact of patient-to-nurse ratios on survival for IHCA patients. The finding that overnight shift increased the mortality risk of IHCA patients in general wards or in both ICU and ER prompts the effort to achieve better work environments and decrease patient-to-nurse ratios in the future.

## Conclusions

IHCA patients, including those in ICUs, ERs, and general wards, who are receiving ventilator support and vasoactive agents had poor survival. Better survival was observed in patients with VT and VF as initial rhythms. However, old age had a negative effect on overall survival. Overnight shifts were associated with poor survival in the general ward subgroup, although no significance was found in the overall cohort and ICU/ER subgroup. Thus, efforts to decrease patient-to-nurse ratios during overnights shift might improve survival in patients after IHCA.

## Data Availability Statement

The original contributions generated for this study are included in the article/supplementary material, further inquiries can be directed to the corresponding author/s.

## Ethics Statement

The studies involving human participants were reviewed and approved by The Institutional Review Board (IRB) of the Kaohsiung Veterans General Hospital approved this study (No. VGHKS19- EM 12-01). Written informed consent was not required for this study as the JCT dataset consists of de-identified secondary data for research purposes.Written informed consent for participation was not required for this study in accordance with the national legislation and the institutional requirements.

## Author Contributions

W-CH: concept and design. S-YW and E-HY: acquisition, analysis, or interpretation of data. M-TW: drafting of the manuscript. W-CH and DY: critical revision of the manuscript for important intellectual content. S-YW and E-HY: statistical analysis. W-CH: administrative, technical, or material support. W-CH and H-HL: supervision. All authors contributed to the article and approved the submitted version.

## Conflict of Interest

The authors declare that the research was conducted in the absence of any commercial or financial relationships that could be construed as a potential conflict of interest.
